# Holistic approaches to living well with endometriosis

**DOI:** 10.12688/f1000research.142586.1

**Published:** 2024-04-23

**Authors:** Jessica Desai, Sophie Strong, Elizabeth Ball

**Affiliations:** 1 Central and North West London NHS Foundation Trust, London, England, UK; 2 Department of Obstetrics and Gynaecology, The Royal London Hospital, Barts Health NHS Trust, London, E1 1FR, UK; 3 Centre for Maternal & Child Health Research, School of Health Sciences, City University of London, UK

**Keywords:** Endometriosis, holistic treatment for endometriosis, complementary treatment for endometriosis, chronic pelvic pain

## Abstract

Endometriosis is a common chronic condition for which there is currently no cure. Those suffering from endometriosis-related pelvic pain (ERPP) may struggle with side effects and/or risks presented by conventional medical and surgical treatment strategies, or not get pain relief. Increasing numbers of endometriosis patients wish to explore holistic management with fewer side effects, however it is important that medical professionals maintain an evidence-based practice for recommended treatments. We present up-to-date evidence of holistic strategies used for managing ERPP including nutritional strategies, body and mind therapies, acupuncture, traditional Chinese medicine (TCM) and the use of adjunct devices such as phallus length reducers and transcutaneous electrical nerve stimulation (TENS).

**Recent findings:**

*Nutrition:* Gluten-free, low-nickel and high intake of omega-3 polyunsaturated fatty acids diets improve ERPP. Low FODMAP (fermentable oligo-, di-, monosaccharides and polyols) is helpful in those with concurrent irritable bowel syndrome. *Body and mind:* Cognitive behaviour therapy (CBT) is particularly beneficial in postoperative pain reduction, whilst mindfulness has been shown to reduce pain scores and dyschezia. Progressive muscle relaxation therapy and regular yoga sessions improve ERPP and Quality of life.

*Acupuncture:* 15 randomised control trials assessing acupuncture and moxibustion show improved pain scores when compared to those receiving conventional therapies alone. *Adjunct devices:* TENS improves deep dyspareunia and lessens the number of days pain is experienced.

**Conclusions:**

Holistic management strategies for ERPP should be incorporated into routine counselling when discussing conservative, medical and or surgical treatments for endometriosis. The growing evidence presented for the use of holistic management strategies gives hope to those patients who cannot have, or don’t respond to conventional approaches and as an adjunct alongside standard treatments.

## Introduction

Endometriosis, an inflammatory women’s health condition affecting about 10% of the female population, ^1^ can cause infertility and chronic pelvic pain (CPP) with acute flares. Pain centralisation is thought to play a role for many patients. ^2^ The mainstay of treatment has been laparoscopic excision or ablation of implants/affected organs. Hormonal or non-hormonal medical therapies can replace or complement this, along with pain relief.

However, in our experience at a busy tertiary endometriosis centre in London, many women with endometriosis-related pelvic pain (ERPP) have adopted holistic approaches to manage pain and improve their quality of life (QOL). Recent developments also call us to reassess and contextualise traditional treatments, to look further afield for more comprehensive approaches, which support patient autonomy and empowerment toward living well with endometriosis.

1. A systematic review (SR) of surgical outcomes for endometriosis ^3^ showed that 11.8% of patients reported no pain improvement. Women with isolated surface endometriosis in particular, may not benefit from surgery, ^4^ which is currently the focus of randomised control trial (RCT) ESPriT2 ( NCT04081532).
2. The coronavirus disease 2019 (COVID-19) pandemic transformed how women seek advice on endometriosis. ‘Hormonophobia’ appears to be on the rise on social media platforms, with increasing numbers of women sharing negative experiences of hormonal contraceptives, reducing the willingness to try them. ^5^
3. Antidepressants and Gabapentin, previously prescribed as neuromodulators are not as effective as previously thought. ^6^

In front of this backdrop and with an understanding that living with chronic conditions can be eased by holistic approaches and self-management, ^7^ we present recent advances in this field.

### Nutrition

Numerous SRs support the role of nutrition in managing chronic pain conditions. ^8^^,^^9^ Optimising diet quality with a high intake of anti-inflammatory nutrients reduces pain severity by modulating the body’s inflammatory response. ^10^ Gut microbiome dysbiosis in inflammatory pain conditions such as endometriosis is hypothesised to cause incorrect immune responses resulting in pain from central sensitisation pathways. Probiotics and FODMAP diets (omitting fermentable oligo-, di-, monosaccharides and polyols), are beneficial in treating visceral pain. ^11^ More research into dietary effects on endometriosis is recommended, ^12^ as many studies to date have small population sizes with heterogeneity between intervention groups.

A SR (one RCT and five observational studies) of low FODMAP, gluten-free and low-nickel diets as well as high intake of omega-3 polyunsaturated fatty acids (average treatment dose palmitoylethanolamide 400 mg & polydatin 40 mg twice daily for 3 months) ^13^^–^^15^ reported that all diets, with the exception of low FODMAP reduced pain. ^16^ Those with concurrent irritable bowel syndrome (IBS) may benefit the most from low-FODMAPs; observational data (n=160) demonstrated symptom improvement compared to patients with IBS alone (72% vs. 40%, respectively, p = 0.001). ^17^

Compared to controls, endometriosis patients may have a higher rate of nickel allergic contact mucositis (odds ratio: 2.474; 95% confidence interval: 1.023~5.988; p = 0.044), ^18^ causing IBS-like symptoms. Reducing nickel-rich foods e.g. tomatoes, whole wheat, and soy, resulted in significant improvement of CPP (p < 0.05) in a prospective 3-month observational study of 31 women with endometriosis and gastrointestinal symptoms. ^19^

Krabbenborg *et al.* ^20^ observed 157 women with endometriosis assessing which dietary modifications patients had already implemented improved their QOL using the EHP-30 score. The most used diets were the endometriosis diet (self-selecting nutrients to omit thought by the individual to worsen their symptoms), gluten free, low-FODMAP, low-lactose and weight loss diets. Although EHP-30 scores did not significantly alter with dietary modification, pain reduction was noted in 71.3% of patients, with gluten-free showing the greatest impact. Dietary modifications have a greater impact with longer adherence.

In a placebo-controlled triple-blind RCT (n = 120), garlic extract (400 mg daily over 12 weeks) showed a significant reduction in ERPP (p < 0.05). Purported mechanisms are reduction in oxidative stresses, prostaglandin production, endometriosis cell proliferation and increased oestrogen elimination. ^21^

Endometriosis severity may be associated with both low and high BMI. ^22^ The association between BMI and endometriosis severity might be more complicated than a simple correlation. A confounding factor for both endometriosis and obesity may be systemic inflammation. ^23^

It is tempting to speculate whether maintenance of a normal BMI is beneficial for symptom control, but studies designed to assess change in weight for ERPP are lacking.

***Body & mind therapies***

Poor mental health may result secondary to the multifactorial impact endometriosis has on physical, sexual, and psychological well-being. ^24^ Strategies such as cognitive behavioural therapy (CBT), yoga and relaxation techniques can be valuable. Increasing evidence suggests psychosocial factors, such as preoperative pain catastrophising independently impact pain experience, severity of symptoms and recurrence of endometriosis. ^25^^,^^26^ Patient awareness and self-uptake of psychological approaches for ERPP are increasingly popular, with 93.8% of women sampled in a cross-sectional survey distributed via The Endometriosis Network Canada (n = 434) utilising at least one psychological management strategy. ^27^

***CBT***

CBT is recognised as an effective treatment for chronic pain and associated mental health conditions, including CPP. ^28^ Current research interest is evidenced by the publication of three RCT protocols assessing efficacy of CBT ^29^^,^^30^ and yoga with CBT ^31^ on QOL of patients with endometriosis. Boersen’s RCT ^30^ aims to recruit 100 patients undergoing endometriosis surgery, assessing benefits of CBT in postoperative care.

Wu *et al.* ^32^ assessed the benefits of CBT plus usual care compared to usual care alone in post-surgical endometriosis patients with a case-control study (Intervention group n = 48, Control group n = 48), utilising one CBT session before and six sessions post-surgery. During a 6-month follow-up, participants provided a score on the depression, anxiety, and stress (DASS-21). Anxiety scores improved significantly (p = 0.0091).

Authors suggest patient education played a large role in self-management of ERPP following CBT.

***Mindfulness***

Mindfulness is a psychological technique that draws on awareness and non-judgemental acceptance of present personal experience. The mindfulness-based stress reduction (MBSR) programme, was first developed by Kabat-Zinn ^33^ as an adjunct to treatment for chronic pain, through relating physical and psychological conditions.

Moreira *et al.* ^34^ performed an RCT to assess the impact of mindfulness on CPP. They adapted the MBSR programme, forming a brief mindfulness-based intervention (bMBI, n = 31, usual care controls n = 32) which had a reduced intensity and reduced duration (4-weeks instead of 8-weeks). Formal meditation was practised around the theme of ‘reconceptualising pain.’ The intervention group showed reduced pain scores & unpleasantness and dyschezia.

Hansen *et al.* ^35^ found that psychological intervention, with a mindfulness focus, did not reduce perception of pain, but did improve QOL in a three-armed RCT. Participants were randomised to three groups: mindfulness and acceptance-based intervention (n = 20), non-specific psychological intervention that did not include mindfulness (relaxation and guided physical therapy) (n = 19), or a waitlist control that included usual treatment (n = 19). All participants received usual treatment which included analgesia. The ten-week programme developed (MY-ENDO), combined Kabat-Zinn’s MBSR programme and acceptance with commitment therapy. There was no statistically significant reduction in ERPP between the MY-ENDO group and non-specific intervention group (p = 0.144, *d* = 0.59). Psychological intervention significantly improved QOL-subscales ‘control and powerlessness’ (p = 0.019, *d* = 0.78), ‘emotional well-being’ (p = 0.003, *d* = 1.01), and ‘social support’ (p = 0.042, *d* = 0.66).

QOL was improved through the positive effects on bowel symptoms, specifically diarrhoea ( *P* = 0.035, *d* = 0.25), within the two intervention groups, thought to be due to physical activity undertaken.

Further studies are needed to determine whether psychological interventions in general improves QOL or whether there is a need for a mindfulness aspect to the intervention.

***Yoga***

Yoga has a long tradition in managing chronic pain. In an AB-design pilot study of 42 women by Ravins *et al.*, ^36^ participants underwent eight-weeks of conventional therapy followed by eight-weeks of 90-minute endometriosis yoga sessions, bi-weekly. EHP-30 scores and numerical pain rating scale were lower following the completion of the yoga sessions (p = 0.001).

Gonçalves’ RCT ^37^ randomly allocated 40 women; an intervention group who practised 90-minutes of yoga bi-weekly for 8 weeks (n = 28) and a control group who did not practise yoga (n = 12). Daily pain was significantly lower among the intervention group (p = 0.0007). EHP-30 domains were assessed at the time of presentation and again at 8-week follow up; pain (p = 0.0046), well-being (p = 0.0009), and image (p = 0.0087) from the central questionnaire, and work (p = 0.0027) and treatment (p = 0.0245) from the modular questionnaire were significantly different. One limitation of this study was the high loss to follow up, with only 57% of participants in the intervention group completing the full yoga-programme, highlighting the challenges faced of adhering to regular yoga practice.

Similar findings were echoed by Saxena *et al.* ^38^ in a randomised case-control study of 60 women with CPP. The intervention group (n = 30) who received yoga therapy with conventional therapy (non-steroidal anti-inflammatory drugs, *NSAIDs*) were compared with the control group (n = 30) who received NSAIDs alone. Pain scores through VAS score and QOL by the World Health Organization quality of life-BREF (WHOQOL-BREF) questionnaire were assessed at the start of the study and again at an 8-week follow up. The yoga-practising group showed a significant decrease in pain intensity (p < 0.001) and improvement in the QOL with a significant increase (p < 0.001) in physical, psychological, social, and environmental domain scores of WHOQOL-BREF.

Enriched environments (consisting of enlarged space, increased physical activity and social interactions) suppresses the development of endometriosis in mice through attenuated adrenergic signalling, enhanced autophagy, and reduced leptin levels. ^39^ Extrapolating this to humans, offering group outdoor physical activities such yoga to optimise environmental enrichment showed significantly less ERPP and perceived stress, improved mood and emotional wellbeing QOL compared with control participants in a recent RCT by Flores. ^40^

***Progressive muscle relaxation (PMR)***

PMR improved anxiety and depression (p < 0.05), and health-related QOL (p < 0.05) for patients with endometriosis in a study of 100 women receiving Gonadotrophin-releasing hormone (GnRH) agonist treatment. Participants were randomly assigned either to a control group or PMR group, who received 12 weeks of PMR training. ^41^

Psychological and physical interventions positively impact on QOL in patients with ERPP. However, there remains a lack of high-powered trials in mind and body therapies. Consideration must be taken for the barriers to accessing psychological interventions. Patients should not feel their pain is less validated if a physiological approach is offered. Smart-phone applications are nowadays suggested to simplify access to Mindfulness. However, those approaches require co-development with stakeholders to be acceptable and used regularly. ^42^

### Physiotherapy

***Pelvic floor muscle physiotherapy***

Pelvic floor muscle dysfunction (specifically levator ani hypertonia and incomplete relaxation) contributes to ERPP with deep infiltrative endometriosis (DIE). ^43^^–^^45^

Pelvic floor physiotherapy (PFP), with 3D/4D trans-perineal ultrasound, increased levator hiatus area (LHA) which in turn improved dyspareunia and pelvic floor muscle relaxation (PFMR) reduced ERPP. Following a successful pilot study, ^46^ Forno *et al.* used trans-perineal ultrasound to assess LHA before and after PFP in an RCT of 34 women. ^47^ Participants were assigned to a no intervention group (n = 17), or the treatment group which involved five PFP sessions (n = 17). Physiotherapy sessions involved the Thiele massage, using digital pressure to elongate and relax muscles, restoring normal tone. PFMR improved on maximum Valsalva manoeuvre in the intervention group compared to the control (20.0 ± 24.8% *vs* –0.5 ± 3.3%, respectively; p = 0.02), and superficial dyspareunia pain scores reduced (p < 0.01).

### Acupuncture

Previous studies have shown acupuncture to be a suitable tool in reducing ERPP, and is considered a safe therapy with minimal side effects. ^48^^,^^49^ Several recent case studies have shown symptomatic improvement with acupuncture. ^50^^,^^51^ Yan *et al.* published a protocol for SR and meta-analysis of RCTs on acupuncture benefits for endometriosis symptoms. ESHRE guidelines ^52^ acknowledge that acupuncture may be a beneficial tool, however the studies that were available at that time were limited and not free from bias.

Wang *et al.* ^53^ recently published a systematic review of 15 RCTs (sample sizes between 10 and 54), which assessed the effectiveness of acupuncture and/or moxibustion for the treatment of endometriosis. Compared with sham acupuncture, actual acupuncture was more effective at reducing dysmenorrhoea VAS pain score (mean difference [MD] − 2.40, 95% CI [− 2.80, − 2.00]; moderate certainty evidence), pelvic pain VAS score (MD − 2.65, 95% CI [− 3.40, − 1.90]; high certainty evidence) and dyspareunia VAS scores (MD − 2.88, [− 3.83, − 1.93]), lessened the size of ovarian cyst (MD − 3.88, 95% CI [− 7.06, − 0.70]), and improved QOL. These promising results suggest that acupuncture is an effective adjunct to treating ERPP.

In a multicentre, randomised, single-blind, placebo-controlled trial ^54^ assessing the effects of acupuncture on endometriosis related symptoms (n = 106), acupuncture was delivered to the intervention group (n = 51) as 30-minute sessions once daily, three times a week, starting one week before expected onset of menstruation, for a total duration of 12-weeks. The control group (n = 53) received sham acupuncture. Lower VAS scores were seen in the intervention group at 12 weeks for dysmenorrhoea (-2.82 (-3.47, -2.18) and QOL, (EHPscore) -18.88 (-31.88, -5.87)), but not for pelvic pain and dyspareunia. At 24 weeks no statistical benefits were seen, suggesting acupuncture is a suitable immediate therapy for endometriosis related dysmenorrhoea, however the effects of acupuncture may not be sustainable over a long period of time and repeated therapy would be necessary.

### Adjunct devices

***Phallus length reducing devices***

The Ohnut© device is a phallus length reducer worn over the penis or penetrating object with the intention to reduce endometriosis-associated deep dyspareunia. The effectiveness of this device is currently being assessed in a pilot RCT of 40 participants by Zhang ^55^ who will be randomised into an intervention group or a waitlist control group.

***Transcutaneous electrical nerve stimulation (TENS)***

A TENS unit passes a current through electrodes placed on the skin for targeted pain relief via the gate control theory. ^56^ Its use has been shown to reduce pain in primary dysmenorrhoea ^57^ and CPP. ^58^^,^^59^

Mira *et al.* ^60^ conducted a multicentre RCT of 101 participants with DIE. The study aimed to identify whether the addition of a TENS unit to hormonal therapy (intervention group; n = 53) would provide a greater therapeutic benefit than hormonal treatment alone (control group; n = 48). The TENS device was used twice a day, 20 minutes per day, for 8 weeks. CPP improved in the intervention group (VAS decreased from 7.11 ± 2.40–4.55 ± 3.08, p < 0.001, 36% decrease), whereas it did not in the control group (VAS from 7.33 ± 2.09–7.06 ± 2.33, p = 0.554, 3.68% decrease). A greater improvement in deep dyspareunia was found in the intervention group, 32.67% reduction vs. 13.84% reduction in the control group. There was a decrease in the number of days participants experienced pain from the first week to the eighth week (from 3.27 to 2.22, p = 0.028, 32.11% decrease), which was not identified in the control group (from 4.55 to 4.07, p = 0.203, 10.54% decrease). This study was conducted over a relatively short time interval, therefore due to the chronic nature of endometriosis, further research is needed to assess whether benefits from TENS units are sustained longer-term.

### Cannabinoid (CBT)

A cross sectional survey ^61^ of 113 women with pelvic, perineal pain, dyspareunia or endometriosis was conducted to gather information regarding patient cannabis use. 26/113 (23%) participants reported cannabis use, of which only 5/26 obtained cannabis through a medical programme, 25 had complete data and were analysed. 15/25 (60%) used a combination of cannabidiol (CBD) and tetrahydrocannabinol (THC). There was no significant difference between the demographics of cannabis users and nonusers. Overall, 24/25 (96%) of participants reported improvement in symptoms such as pain, depression and sleep disturbance with the use of cannabis. It is important to note that participants from both groups also utilised alternative medications and therapies, and therefore reported symptom improvement cannot be confidently solely attributed to cannabis use.

***Traditional Chinese medicine (TCM)***

Zhao *et al.* ^62^ performed a non-blinded RCT of 320 patients undergoing endometriosis surgery to investigate the effects of TCM (activating blood circulation and removing blood stasis treatment based on syndrome differentiation; n = 131) and Western medicine (GnRH agonist or progesterones; n = 141) on QOL postoperatively.

Pre-treatment WHOQOL-BREF scores, a QOL assessment tool with four domains including physical health, psychological, social relationships and environment, showed no significant difference between the two groups (p > 0.05), however post-treatment scores in the TCM group were significantly improved (p < 0.05) and the scores of 4 items (mobility, activities of daily living, sexual activity, QOL score) were also statistically significantly better (p < 0.05).

Flower *et al.* ^63^ published a Cochrane review assessing the effects of Chinese herbal medicine (CHM) for endometriosis, however only 2 RCTs were included (n = 158), neither of which assessed CHM vs. placebo. The first showed no significant different in ERPP between CHM and gestrinone administration post laparoscopic treatment (95.65% vs. 93.87%; risk ratio (RR) 1.02, 95% confidence interval (CI) 0.93 to 1.12, one RCT). Combined oral CHM and herbal enema provided better improvement in dysmenorrhoea than with danazol (RR 5.06, 95% CI 1.28 to 20.05; RR 5.63, 95% CI 1.47 to 21.54, respectively). There was no significant difference in lumbosacral pain, rectal discomfort, or vaginal nodule tenderness between CHM and danazol. Flower reports a paucity of robust studies assessing the effects of CHM and that the current studies available have been too small to apply statistical analysis.

## Discussion

The previous cornerstones of endometriosis care have been shaken. Neuromodulators are less effective than assumed, ^6^ a meaningful proportion do not get pain relief from surgery ^3^ and ⅓ do not respond to progesterone. Complementary, self-management and lifestyle approaches are moving from fringe interest into mainstream endometriosis care.

A historic RCT ^64^ has shown multimodal holistic approaches yield superior outcomes to early laparoscopy in CPP.

Current endometriosis centre accreditation weights bowel surgery heavily but patient education and signposting to holistic evidence-based care is left to enthusiastic HCPs, specialist nurses and patient charities, resulting in care inequities. Accreditation hinges on a multidisciplinary team of surgeons/urologists, but not with pelvic pain physiotherapists, nutritionists and psychologists.

Numerous calls for more research into complementary approaches need to be answered by appropriate funding.

Within a patient journey, complementary approaches could be used in the following models as a primary approach or in conjunction with routine treatment. ^4^

1. Future women’s health hubs can identify DIE, likely to respond to surgery with specialised scanning even before referral to secondary and tertiary care. Models initiating this in the community would improve patient journeys and shorten the delay in endometriosis patients accessing care.
2. Peri-operatively in the context of pre- and rehabilitation: surgery should no longer be seen in isolation but embedded in education and self-care. Clinicians observe patients recover faster and better from endometriosis surgery if they go into surgery having practised pre-habilitation.
3. An adjunct to hormonal, surgical and pain-relieving western approaches.
4. In the future, complementary and self-care techniques may be used in prevention of disease recurrence, whereas today the only evidence base is in hormonal manipulation ^65^ but future evidence may enable clinicians to recommend preventive approaches.

## Data Availability

No data are associated with this article.

## References

[1] Smolarz B Szyłło K Romanowicz H : Endometriosis: Epidemiology, Classification, Pathogenesis, Treatment and Genetics (Review of Literature). *Int. J. Mol. Sci.* 2021;22(19). 10.3390/ijms221910554 34638893 PMC8508982

[2] Zheng P Zhang W Leng J : Research on central sensitization of endometriosis-associated pain: a systematic review of the literature. *J. Pain Res.* 2019;12:1447–1456. 10.2147/JPR.S197667 31190954 PMC6514255

[3] Singh SS Gude K Perdeaux E : Surgical Outcomes in Patients With Endometriosis: A Systematic Review. *J. Obstet. Gynaecol. Can.* 2020;42(7):881–888.e11. 10.1016/j.jogc.2019.08.004 31718952

[4] Ball E Karavadra B Kremer-Yeatman BJ : Systematic review of patient-specific pre-operative predictors of pain improvement to endometriosis surgery. *Reprod. Fertil.* 2021;2(1):69–80. 10.1530/RAF-20-0057 35128434 PMC8812445

[5] Le Guen M Schantz C Régnier-Loilier A : Reasons for rejecting hormonal contraception in Western countries: A systematic review. *Soc. Sci. Med.* 2021;284:114247. 10.1016/j.socscimed.2021.114247 34339927

[6] Vincent K Baranowski A Bhattacharya S : GaPP2, a multicentre randomised controlled trial of the efficacy of gabapentin for the management of chronic pelvic pain in women: study protocol. *BMJ Open.* 2018;8(1):e014924. 10.1136/bmjopen-2016-014924 29391360 PMC5879736

[7] Koirala B Peeler A Dennison Himmelfarb C : Living with multiple chronic conditions: How we achieve holistic care and optimize health outcomes. *J. Adv. Nurs.* 2023;79(2):e7–e9. 10.1111/jan.15433 36062872 PMC9877113

[8] Field R Pourkazemi F Turton J : Dietary Interventions Are Beneficial for Patients with Chronic Pain: A Systematic Review with Meta-Analysis. *Pain Med.* 2021;22(3):694–714. 10.1093/pm/pnaa378 33202007

[9] Genel F Kale M Pavlovic N : Health effects of a low-inflammatory diet in adults with arthritis: a systematic review and meta-analysis. *J. Nutr. Sci.* 2020;9:e37. 10.1017/jns.2020.31 32983422 PMC7503186

[10] Brain K Burrows TL Bruggink L : Diet and Chronic Non-Cancer Pain: The State of the Art and Future Directions. *J. Clin. Med.* 2021;10(21). 10.3390/jcm10215203 34768723 PMC8584994

[11] Ustianowska K Ustianowski Ł Machaj F : The Role of the Human Microbiome in the Pathogenesis of Pain. *Int. J. Mol. Sci.* 2022;23(21). 10.3390/ijms232113267 36362056 PMC9659276

[12] Nirgianakis K Egger K Kalaitzopoulos DR : Effectiveness of Dietary Interventions in the Treatment of Endometriosis: a Systematic Review. *Reprod. Sci.* 2022;29(1):26–42. 10.1007/s43032-020-00418-w 33761124 PMC8677647

[13] Cobellis L Castaldi MA Giordano V : Effectiveness of the association micronized N-Palmitoylethanolamine (PEA)-transpolydatin in the treatment of chronic pelvic pain related to endometriosis after laparoscopic assessment: a pilot study. *Eur. J. Obstet. Gynecol. Reprod. Biol.* 2011;158(1):82–86. 10.1016/j.ejogrb.2011.04.011 21601979

[14] Giugliano E Cagnazzo E Soave I : The adjuvant use of N-palmitoylethanolamine and transpolydatin in the treatment of endometriotic pain. *Eur. J. Obstet. Gynecol. Reprod. Biol.* 2013;168(2):209–213. 10.1016/j.ejogrb.2013.01.009 23415738

[15] Indraccolo U Barbieri F : Effect of palmitoylethanolamide-polydatin combination on chronic pelvic pain associated with endometriosis: preliminary observations. *Eur. J. Obstet. Gynecol. Reprod. Biol.* 2010;150(1):76–79. 10.1016/j.ejogrb.2010.01.008 20176435

[16] Sverrisdóttir U Hansen S Rudnicki M : Impact of diet on pain perception in women with endometriosis: A systematic review. *Eur. J. Obstet. Gynecol. Reprod. Biol.* 2022;271:245–249. 10.1016/j.ejogrb.2022.02.028 35245715

[17] Moore JS Gibson PR Perry RE : Endometriosis in patients with irritable bowel syndrome: Specific symptomatic and demographic profile, and response to the low FODMAP diet. *Aust. N. Z. J. Obstet. Gynaecol.* 2017;57(2):201–205. 10.1111/ajo.12594 28303579

[18] Yuk JS Shin JS Shin JY : Nickel Allergy Is a Risk Factor for Endometriosis: An 11-Year Population-Based Nested Case-Control Study. *PLoS One.* 2015;10(10):e0139388. 10.1371/journal.pone.0139388 26439741 PMC4594920

[19] Borghini R Porpora MG Casale R : Irritable Bowel Syndrome-Like Disorders in Endometriosis: Prevalence of Nickel Sensitivity and Effects of a Low-Nickel Diet. An Open-Label Pilot Study. *Nutrients.* 2020;12(2). 10.3390/nu12020341 32012984 PMC7071203

[20] Krabbenborg I Roos N de Grinten P van der : Diet quality and perceived effects of dietary changes in Dutch endometriosis patients: an observational study. *Reprod. Biomed. Online.* 2021;43(5):952–961. 10.1016/j.rbmo.2021.07.011 34493462

[21] Amirsalari S Behboodi Moghadam Z Taghizadeh Z : The Effect of Garlic Tablets on the Endometriosis-Related Pains: A Randomized Placebo-Controlled Clinical Trial. *Evid. Based Complement. Alternat. Med.* 2021;2021:5547058. 34335819 10.1155/2021/5547058 PMC8315864

[22] Liu Y Zhang W : Association between body mass index and endometriosis risk: a meta-analysis. *Oncotarget.* 2017;8(29):46928–46936. 10.18632/oncotarget.14916 28159926 PMC5564533

[23] Hong J Yi KW : What is the link between endometriosis and adiposity? *Obstet. Gynecol. Sci.* 2022;65(3):227–233. 10.5468/ogs.21343 35081675 PMC9119730

[24] Netzl J Gusy B Voigt B : Chronic Pelvic Pain in Endometriosis: Cross-Sectional Associations with Mental Disorders, Sexual Dysfunctions and Childhood Maltreatment. *J. Clin. Med.* 2022;11(13). 10.3390/jcm11133714 PMC9267229 35807000

[25] McPeak AE Allaire C Williams C : Pain Catastrophizing and Pain Health-Related Quality-of-Life in Endometriosis. *Clin. J. Pain.* 2018;34(4):349–356. 10.1097/AJP.0000000000000539 28731958

[26] Carey ET Martin CE Siedhoff MT : Biopsychosocial correlates of persistent postsurgical pain in women with endometriosis. *Int. J. Gynaecol. Obstet.* 2014;124(2):169–173. 10.1016/j.ijgo.2013.07.033 24290537

[27] Gholiof M Adamson-De Luca E Foster WG : Prevalence of Use and Perceived Effectiveness of Medical, Surgical, and Alternative Therapies for Endometriosis Pain in Canadians. *J. Obstet. Gynaecol. Can.* 2023;45(1):11–20. 10.1016/j.jogc.2022.11.003 36455861

[28] Brooks T Sharp R Evans S : Psychological Interventions for Women with Persistent Pelvic Pain: A Survey of Mental Health Clinicians. *J. Multidiscip. Healthc.* 2021;14:1725–1740. 10.2147/JMDH.S313109 34262286 PMC8275108

[29] Schubert K Lohse J Kalder M : Internet-based cognitive behavioral therapy for improving health-related quality of life in patients with endometriosis: study protocol for a randomized controlled trial. *Trials.* 2022;23(1):300. 10.1186/s13063-022-06204-0 35414092 PMC9006397

[30] Boersen Z Oosterman J Hameleers EG : Determining the effectiveness of cognitive behavioural therapy in improving quality of life in patients undergoing endometriosis surgery: a study protocol for a randomised controlled trial. *BMJ Open.* 2021;11(12):e054896. 10.1136/bmjopen-2021-054896 34880026 PMC8655560

[31] Mikocka-Walus A Druitt M O’Shea M : Yoga, cognitive-behavioural therapy versus education to improve quality of life and reduce healthcare costs in people with endometriosis: a randomised controlled trial. *BMJ Open.* 2021;11(8):e046603. 10.1136/bmjopen-2020-046603 34373298 PMC8354255

[32] Wu S Wang X Liu H : Efficacy of cognitive behavioral therapy after the surgical treatment of women with endometriosis: A preliminary case-control study. *Medicine (Baltimore).* 2022;101(51):e32433. 10.1097/MD.0000000000032433 36595829 PMC9794269

[33] Noonan S : Mindfulness-based stress reduction. *Can. Vet. J.* 2014;55(2):134–135. 24489390 PMC3894868

[34] Moreira MF Gamboa OL Pinho Oliveira MA : A single-blind, randomized, pilot study of a brief mindfulness-based intervention for the endometriosis-related pain management. *Eur. J. Pain.* 2022;26(5):1147–1162. 10.1002/ejp.1939 35276031

[35] Hansen KE Brandsborg B Kesmodel US : Psychological interventions improve quality of life despite persistent pain in endometriosis: results of a 3-armed randomized controlled trial. *Qual. Life Res.* 2023;32(6):1727–1744. 10.1007/s11136-023-03346-9 36797461 PMC10172241

[36] Ravins I Joseph G Tene L : The Effect of Practicing “Endometriosis Yoga” on Stress and Quality of Life for Women with Endometriosis: AB Design Pilot Study. *Altern. Ther. Health Med.* 2023;29(3):8–14. 35839113

[37] Gonçalves AV Barros NF Bahamondes L : The Practice of Hatha Yoga for the Treatment of Pain Associated with Endometriosis. *J. Altern. Complement. Med.* 2017;23(1):45–52. 10.1089/acm.2015.0343 27869485

[38] Saxena R Gupta M Shankar N : Effects of yogic intervention on pain scores and quality of life in females with chronic pelvic pain. *Int. J. Yoga.* 2017;10(1):9–15. 10.4103/0973-6131.186155 28149062 PMC5225749

[39] Yin B Jiang H Liu X : Enriched Environment Decelerates the Development of Endometriosis in Mouse. *Reprod. Sci.* 2020;27(7):1423–1435. 10.1007/s43032-019-00117-1 32318984

[40] I F, B B-C, G DH, D R-S, V L-R, C N-V : Impact of Environmental Enrichment on Pain, Perceived Stress, Mental Health and Quality of Life of Women with Endometriosis The World Congress on Endometriosis; Edinburgh, UK. 2023.

[41] Zhao L Wu H Zhou X : Effects of progressive muscular relaxation training on anxiety, depression and quality of life of endometriosis patients under gonadotrophin-releasing hormone agonist therapy. *Eur. J. Obstet. Gynecol. Reprod. Biol.* 2012;162(2):211–215. 10.1016/j.ejogrb.2012.02.029 22455972

[42] Ball E Rivas C : Health Apps Require Co-development to Be Acceptable and Effective. *Front. Psychol.* 2021;12:714453. 10.3389/fpsyg.2021.714453 34335428 PMC8322940

[43] Fraga MV Oliveira Brito LG Yela DA : Pelvic floor muscle dysfunctions in women with deep infiltrative endometriosis: An underestimated association. *Int. J. Clin. Pract.* 2021;75(8):e14350. 10.1111/ijcp.14350 33973308

[44] Silva JP da Almeida BM de Ferreira RS : Sensory and muscular functions of the pelvic floor in women with endometriosis - cross-sectional study. *Arch. Gynecol. Obstet.* 2023;308(1):163–170. 10.1007/s00404-023-07037-1 37042996

[45] Arena A Degli Esposti E Cocchi L : Three-Dimensional Ultrasound Evaluation of Pelvic Floor Muscle Contraction in Women Affected by Deep Infiltrating Endometriosis: Application of a Quick Contraction Scale. *J. Ultrasound Med.* 2022;41(12):2973–2979. 10.1002/jum.15996 35532292

[46] Del Forno S Arena A Alessandrini M : Transperineal Ultrasound Visual Feedback Assisted Pelvic Floor Muscle Physiotherapy in Women With Deep Infiltrating Endometriosis and Dyspareunia: A Pilot Study. *J. Sex Marital Ther.* 2020;46(7):603–611. 10.1080/0092623X.2020.1765057 32579077

[47] Del Forno S Arena A Pellizzone V : Assessment of levator hiatal area using 3D/4D transperineal ultrasound in women with deep infiltrating endometriosis and superficial dyspareunia treated with pelvic floor muscle physiotherapy: randomized controlled trial. *Ultrasound Obstet. Gynecol.* 2021;57(5):726–732. 10.1002/uog.23590 33428320

[48] Mira TAA Buen MM Borges MG : Systematic review and meta-analysis of complementary treatments for women with symptomatic endometriosis. *Int. J. Gynaecol. Obstet.* 2018;143(1):2–9. 10.1002/ijgo.12576 29944729

[49] Payne JA : *Acupuncture for Endometriosis: A Case Study. Med Acupunct.* 31. United States: Copyright 2019, Mary Ann Liebert, Inc., Publishers;2019; pp.392–394. 10.1089/acu.2019.1379 PMC6918512 31871528

[50] Martin BR : Multimodal Care for Headaches, Lumbopelvic Pain, and Dysmenorrhea in a Woman With Endometriosis: A Case Report. *J. Chiropr. Med.* 2021;20:148–157. 20. United States: © 2022 by National University of Health Sciences. 10.1016/j.jcm.2021.10.002 35463839 PMC9023129

[51] Yan Q Li J Zeng J : The role of acupuncture in the treatment of women with pain in endometriosis: A protocol for systematic review and meta-analysis. *Medicine (Baltimore).* 2021;100(49):e27582. 10.1097/MD.0000000000027582 34889220 PMC8663820

[52] Becker CM Bokor A Heikinheimo O : ESHRE guideline: endometriosis. *Hum. Reprod. Open.* 2022;2022(2):hoac009. 10.1093/hropen/hoac009 35350465 PMC8951218

[53] Wang Y Coyle ME Hong M : Acupuncture and moxibustion for endometriosis: A systematic review and analysis. *Complement. Ther. Med.* 2023;76:102963. 10.1016/j.ctim.2023.102963 37453585

[54] Li PS Peng XM Niu XX : Efficacy of acupuncture for endometriosis-associated pain: a multicenter randomized single-blind placebo-controlled trial. *Fertil. Steril.* 2023;119(5):815–823. 10.1016/j.fertnstert.2023.01.034 36716811

[55] Zhang SXJ MacLeod RGK Parmar G : Ohnut Versus a Waitlist Control for the Self-management of Endometriosis-Associated Deep Dyspareunia: Protocol for a Pilot Randomized Controlled Trial. *JMIR Res. Protoc.* 2023;12:e39834. 10.2196/39834 36972117 PMC10131731

[56] Teoli D An J : Transcutaneous Electrical Nerve Stimulation. *StatPearls.* Treasure Island (FL): StatPearls Publishing. Copyright © 2023, StatPearls Publishing LLC;2023. 30725873

[57] Arik MI Kiloatar H Aslan B : The effect of TENS for pain relief in women with primary dysmenorrhea: A systematic review and meta-analysis. *Explore (N.Y.).* 2022;18(1):108–113. 10.1016/j.explore.2020.08.005 32917532

[58] Bridger C Prabhala T Dawson R : Neuromodulation for Chronic Pelvic Pain: A Single-Institution Experience With a Collaborative Team. *Neurosurgery.* 2021;88(4):819–827. 10.1093/neuros/nyaa537 33372201 PMC7956019

[59] Guy M Foucher C Juhel C : Transcutaneous electrical neurostimulation relieves primary dysmenorrhea: A randomized, double-blind clinical study versus placebo. *Prog. Urol.* 2022;32(7):487–497. 10.1016/j.purol.2022.01.005 35249825

[60] Mira TAA Yela DA Podgaec S : Hormonal treatment isolated versus hormonal treatment associated with electrotherapy for pelvic pain control in deep endometriosis: Randomized clinical trial. *Eur. J. Obstet. Gynecol. Reprod. Biol.* 2020;255:134–141. 10.1016/j.ejogrb.2020.10.018 33129015

[61] Carrubba AR Ebbert JO Spaulding AC : Use of Cannabis for Self-Management of Chronic Pelvic Pain. *J. Womens Health (Larchmt).* 2021;30(9):1344–1351. 10.1089/jwh.2020.8737 33252316

[62] Zhao RH Liu Y Tan Y : Chinese medicine improves postoperative quality of life in endometriosis patients: a randomized controlled trial. *Chin. J. Integr. Med.* 2013;19(1):15–21. 10.1007/s11655-012-1196-6 23275012

[63] Flower A Liu JP Lewith G : Chinese herbal medicine for endometriosis. *Cochrane Database Syst. Rev.* 2012;2012(5):CD006568. 10.1002/14651858.CD006568.pub3 PMC12817023 22592712

[64] Peters AA Dorst E van Jellis B : A randomized clinical trial to compare two different approaches in women with chronic pelvic pain. *Obstet. Gynecol.* 1991;77(5):740–744. 1826544

[65] Zheng Q Mao H Xu Y : Can postoperative GnRH agonist treatment prevent endometriosis recurrence? A meta-analysis. *Arch. Gynecol. Obstet.* 2016;294(1):201–207. 10.1007/s00404-016-4085-y 27052442

