# Holistic approaches to living well with endometriosis

**DOI:** 10.12688/f1000research.142586.2

**Published:** 2024-11-08

**Authors:** Jessica Desai, Sophie Strong, Elizabeth Ball

**Affiliations:** 1 Central and North West London NHS Foundation Trust, London, England, UK; 2 Department of Obstetrics and Gynaecology, The Royal London Hospital, Barts Health NHS Trust, London, E1 1FR, UK; 3 Centre for Maternal & Child Health Research, School of Health Sciences, City University of London, UK

**Keywords:** Endometriosis, holistic treatment for endometriosis, complementary treatment for endometriosis, chronic pelvic pain

## Abstract

Endometriosis is a common chronic non curable neuro-inflammatory condition, which can cause endometriosis-related pelvic pain (ERPP). Sufferers may struggle with side effects and/or risks from conventional medical and surgical treatments, or not get pain relief. Increasing numbers of endometriosis patients wish to explore holistic management with fewer side effects, however it is important that medical professionals maintain an evidence-based practice for recommended treatments. We present up-to-date evidence of holistic strategies used for managing ERPP including nutrition, body and mind therapies, acupuncture, traditional Chinese medicine (TCM) and the use of adjunct devices such as phallus length reducers and transcutaneous electrical nerve stimulation (TENS).

**Recent findings:**

**Nutrition:** Gluten-free, low-nickel and high intake of omega-3 polyunsaturated fatty acids diets improve ERPP. Low FODMAP (fermentable oligo-, di-, monosaccharides and polyols), plant-based diet and antioxidant vitamin supplementation is helpful including those with concurrent irritable bowel syndrome.

**Body and Mind:** Cognitive behaviour therapy (CBT) is beneficial in postoperative pain reduction, whilst mindfulness has been shown to reduce pain scores and dyschezia. Progressive muscle relaxation therapy and regular yoga sessions improve ERPP and Quality of life.

**Acupuncture:** Acupuncture and moxibustion show improved pain scores compared to conventional therapies alone.

**Adjunct devices:** TENS improves deep dyspareunia and reduces the number of days pain is experienced.

**Summary:**

Holistic management strategies for ERPP should be incorporated into routine counselling when discussing conservative, medical and or surgical treatments for endometriosis. The growing evidence presented for the use of holistic management strategies gives hope to those patients who cannot have, or don’t respond to conventional approaches and as an adjunct alongside standard treatments. These findings should be incorporated into the routine counselling when seeing patients in the gynaecology outpatient setting presenting with chronic pelvic pain.

## Introduction

Endometriosis, an inflammatory women’s disease affecting about 10% of the female population, ^1^ can cause infertility and chronic pelvic pain (CPP) with pain centralisation for many patients. ^2^ The mainstay of treatment has been laparoscopic removal of endometriosis. Hormonal or non-hormonal medication and pain relief can replace or complement this.

However, in our tertiary endometriosis centre, many women with ERPP have adopted holistic approaches to manage pain and improve quality of life (QoL). Recent developments call us to reassess and contextualise traditional treatments, and to look for comprehensive approaches, which support patient autonomy and empowerment toward living well with endometriosis.

1. In a systematic review (SR) of surgical outcomes for endometriosis, ^3^ 11.8% of patients reported no pain improvement. Women with isolated surface endometriosis in particular may not benefit from surgery, ^4^ which is currently investigated ESPriT2 ( NCT04081532). ^5^
2. Postoperative functional impairment after laparoscopy for endometriosis include voiding problems and urinary tract infections. ^6^ Long term functional bowel impairment is associated with colorectal endometriosis surgery. ^7^
3. Since the Covid-19 pandemic ‘Hormone-phobia’ is on the rise on social media platforms, with women sharing negative experiences of hormonal contraceptives, reducing the willingness to try them. ^8^
4. Antidepressants and Gabapentin, previously prescribed as neuromodulators in chronic pain are not as effective as previously thought. ^9^^,^^10^

Given the above and understanding that living with chronic conditions can be eased by holistic approaches and self-management, ^11^ we present recent advances.

### Inclusion and exclusion criteria

All studies reporting the assessment and outcomes of selected holistic management for pelvic pain were included (randomised and non-randomised controlled trials, cohort studies and case series).

### Search methodology

We performed a PubMed search with terms to include holistic management strategies for endometriosis, including the search terms applied of: endometriosis with nutrition, diet, cognitive behavioural therapy, mindfulness, yoga, progressive muscle relaxation, physiotherapy, acupuncture, devices, TENS, cannabis, Chinese medicine.

We did not perform formal risk of bias assessment, but reported bias risks like high attrition rates for individual studies.

### Nutrition

The role of nutrition in managing chronic pain conditions is investigated in two SRs. ^12^^,^^13^ A high intake of anti-inflammatory nutrients reduces pain severity by modulating inflammation. ^14^ Gut microbiome dysbiosis is hypothesised to cause incorrect immune responses resulting in pain from central sensitisation pathways in inflammatory conditions such as endometriosis. Probiotics and FODMAP diets (omitting fermentable oligo-, di-, monosaccharides and polyols), are beneficial in treating visceral pain. ^15^ More research into diet in endometriosis is recommended, ^16^ given small population sizes with heterogeneity between intervention groups.

One SR (one RCT and five observational studies) of low FODMAP, gluten-free and low-nickel diets as well as high intake of omega-3 polyunsaturated fatty acids (average treatment dose palmitoylethanolamide 400 mg & polydatin 40 mg twice daily for 3 months) ^17^^–^^19^ reported that all diets, with the exception of low FODMAP reduced pain. ^20^ However, those with endometriosis and irritable bowel syndrome (IBS) may benefit from low-FODMAPs; observational data (n=160) demonstrated symptom improvement compared to patients with IBS alone (72% vs. 40%, respectively, *P*=0.001). ^21^

Interestingly, women with endometriosis are approximately three times more likely to develop IBS, ^22^ posing diagnostic challenges since symptoms (bloating and diarrhoea) may overlap. Velho et al reviews the benefits of anti-inflammatory (e.g. plant based) diets and antioxidant vitamins supplements including vitamin D. ^23^

Compared to controls, endometriosis patients appear to have more allergic nickel contact mucositis (odds ratio: 2.474; 95% confidence interval: 1.023~5.988; *P*=0.044), ^24^ causing IBS-like symptoms. Reducing nickel-rich foods e.g. tomatoes, whole wheat, and soy, resulted in improvement of CPP ( *P*<0.05) in a prospective 3-month observational study of 31 endometriosis patients with gastrointestinal symptoms. ^25^

Krabbenborg et al ^26^ observed 157 endometriosis patients asking which of their own dietary modifications had improved their QoL using the EHP-30 score. The commonest diets were the endometriosis diet (omitting foods that appeared to worsen symptoms), gluten free, low-FODMAP, low-lactose and weight loss diets. Although EHP-30 scores did not significantly alter with dietary modification, pain reduction was noted in 71.3% of patients, with gluten-free showing the greatest impact. Dietary modifications have a greater impact with longer adherence.

In a placebo-controlled triple-blind RCT (n=120) garlic extract (400 mg daily over 12 weeks) showed a significant reduction in ERPP ( *P*<0.05). Purported mechanisms are reduction in oxidative stresses, prostaglandin production, endometriosis cell proliferation and increased oestrogen elimination. ^27^

Both low and high BMI appear to be associated with endometriosis severity, ^28^ a confounding factor for both endometriosis and obesity being systemic inflammation. ^29^

It is tempting to speculate whether maintaining a normal BMI is beneficial for ERPP, and further studies are needed.

### Body & mind therapies

Poor mental health may be a result of the impact endometriosis has on physical, sexual, and psychological well-being. ^30^ Strategies such as cognitive behavioural therapy (CBT), yoga and relaxation techniques can be valuable. Increasing evidence suggests psychosocial factors, such as preoperative pain catastrophising independently impact pain experience, severity of symptoms and recurrence of endometriosis. ^31^^,^^32^ Patient awareness and self-uptake of psychological approaches for ERPP are increasingly popular, with 93.8% of women sampled in a cross-sectional survey distributed via The Endometriosis Network Canada (n=434) utilising at least one psychological management strategy. ^33^

Please see Table 1 for a summary of the body and mind therapies.

**Table 1.** Overview of evidence presented for the different body and mind holistic approaches used for managing ERPP.

Body & Mind therapies			
	Type of study	Sample size	Significant findings
Gholiof et al, 2023	Cross-sectional online survey	434	- 93.8% of respondents reported use of at least one alternative therapy in the past 6 months for ERPP
Wu et al, 2022	Case-control study	96	- CBT combined with usual care in post-surgical endometriosis patients decreased the DASS-21 scores for depression, anxiety and stress in both the study and controls - For anxiety, postintervention DASS-21 score in cases was significantly decreased compared to the controls ( *P*=0.0091).
Moreira et al, 2022	Randomised controlled trial	63	- bMBI showed reduced pain scores, unpleasantness and dychezia in the intervention group
Hansen et al, 2023	Randomised controlled trial	58	- The MY-ENDO programme (combination of MBSR and acceptance with commitment therapy) did not significantly reduce ERPP - Psychological intervention however did significantly improve the specific QoL-subscales ‘control and powerlessness’ ( *P*=10.019, *d*=0.78), ‘emotional wellbeing’ ( *P*=0.003, *d*=1.01) and ‘social support’ ( *P*=0.042, *d*=0.66)
Ravins et al, 2023	AB-design pilot	42	- 8-weeks of 90-minute endometriosis yoga sessions, bi-weekly following 8 weeks of conventional therapy, reduced EHP-30 scores and numerical pain rating scale
Gonçalves et al, 2017	Randomised controlled trial	40	- Daily pain was found to be significantly lower in women who practiced 90-minutes of yoga bi-weekly for 8 weeks ( *P*=0.0007) - Notably 43% of participants in the intervention group did not complete the yoga programme
Saxena et al, 2017	Randomised case-control study	60	- Yoga therapy with NSAID use showed significant reduction in pain intensity ( *P*<0.001) and significant improvement in QOL ( *P*<0.001) compared to NSAID use alone
Zhao et al, 2012		100	- 12 weeks of PMR training improved anxiety and depression ( *P*<0.05), and health related QoL ( *P*<0.05) for women with endometriosis receiving GnRH agonist treatment

ERPP: endometriosis related pelvic pain; CBT: cognitive behavioural therapy; DASS-21: Depression, Anxiety and Stress Scale - 21; bMBI: brief mindfulness based intervention; MY-ENDO: Mind Your ENDOmetriosis; MBSR: Mindfulness Based Stress Reduction; QoL: quality of life; EHP-30: endometriosis health profile – 30; NSAID: non-steroidal anti-inflammatory; PMR: progressive muscle relaxation; GnRH: gonadotrophin releasing hormone.

***CBT***

CBT is recognised as an effective treatment for chronic pain and associated mental health conditions, including CPP. ^34^ Three recent RCT protocols assessing efficacy of CBT ^35^^,^^36^ and yoga with CBT ^37^ on QoL of patients with endometriosis indicate current interest. Boersen’s RCT ^36^ aims to assess CBT in 100 postoperative endometriosis patients.

Wu et al ^38^ assessed CBT with usual care compared to usual care alone in post-surgical endometriosis patients in a case-control study (Interventions n=48, Controls n=48), utilising one CBT session before and six sessions post-surgery. During a 6-month follow-up, participants scored on the depression, anxiety, and stress scale (DASS-21). Anxiety scores improved significantly ( *P*=.0091).

Authors highlight the important role of patient education in self-management of ERPP following CBT.

***Mindfulness***

Mindfulness is a psychological technique that draws on awareness and non-judgemental acceptance of present personal experience. The mindfulness-based stress reduction (MBSR) programme, developed by Kabat-Zinn ^39^ is an adjunct to treatment for chronic pain, through relating physical and psychological conditions.

Moreira et al ^40^ assessed the impact of mindfulness on ERPP in an RCT: They adapted the MBSR programme, forming a brief mindfulness-based intervention (bMBI, n=31, usual care controls n=32) with reduced intensity and duration (4-weeks instead of 8-weeks). Formal meditation was practised around the theme of ‘reconceptualising pain.’ The intervention group showed reduction in pain scores, pain unpleasantness and dyschezia.

Hansen et al ^41^ found that psychological intervention, improved QoL in a three-armed RCT, without reducing ERPP perception. Endometriosis patients were randomised to three groups: mindfulness and acceptance-based intervention (n=20), non-specific psychological intervention without mindfulness (relaxation and guided physical therapy) (n=19), or waitlist control with usual treatment only (n=19). All participants received usual treatment including analgesia. The ten-week programme (MY-ENDO) combined Kabat-Zinn’s MBSR programme and acceptance and commitment therapy (ACT). There was no significant reduction in ERPP between the MY-ENDO and non-specific psychological intervention ( *P*=0.144, *d*=0.59). Psychological intervention significantly improved QoL-subscales ‘control and powerlessness’ ( *P*=0.019, *d*=0.78), ‘emotional well-being’ ( *P*=0.003, *d*=1.01), and ‘social support’ ( *P*=0.042, *d*=0.66).

QoL was improved through the positive effects on bowel symptoms, specifically diarrhoea ( *P*=0.035, *d*=0.25), within the two intervention groups, likely due to physical activity undertaken.

Further studies are needed to determine whether psychological interventions in general improve QoL or whether it is the mindfulness intervention.

***Yoga***

Yoga has a long tradition in managing chronic pain. In an AB-design pilot study (patients served as their own control group) 42 endometriosis patients by Ravins et al, ^42^ participants underwent eight-weeks of conventional therapy followed by eight-weeks of 90-minute endometriosis yoga sessions, bi-weekly. EHP-30 scores and numerical pain rating scale were lower after the yoga sessions ( *P*=0.001).

Gonçalves’ RCT, ^43^ randomised 40 women with ERPP to 90-minutes of yoga bi-weekly for 8 weeks (n=28) or no yoga (n=12). Daily pain was significantly lowered by yoga ( *P*=0.0007). EHP-30 domains were assessed at the time of presentation and at 8-weeks; scores for pain ( *P*=0.0046), well-being ( *P*=0.0009), and self-image ( *P*=0.0087) improved significantly over time only in the yoga group. Only 57% of participants in the intervention group completed the yoga programme, highlighting the challenges faced of adhering to regular yoga practice.

Saxena et al ^44^ also demonstrated benefits of yoga over conventional care; 30 women with CPP were randomised to yoga therapy and 30 to conventional therapy (non-steroidal anti-inflammatory painkillers (NSAIDs). Pain scores (VAS score) and QoL (World Health Organization WHOQOL-BREF questionnaire) were assessed at baseline and 8-weeks. In contrast to the controls the yoga group showed a significant decrease in pain intensity ( *P*<0.001) and QoL improvement with a significant increase ( *P*<0.001) in physical, psychological, social, and environmental domain scores of WHOQOL-BREF.

Enriched environments (more space to move about, increased physical activity and social interactions) suppresses the development of endometriosis in mice by attenuating adrenergic signalling, enhancing autophagy, and reducing leptin levels. ^45^ Extrapolating this to humans, Flores et al ^46^ reported a significant reduction in pelvic pain, perceived stress and improved mood and emotional wellbeing in endometriosis patients who were randomised to outdoor physical activities such group yoga to optimise environmental enrichment as compared to controls.

***Progressive muscle relaxation (PMR)***

PMR is an exercise that reduces stress and anxiety through slowly tensing and relaxing muscle groups throughout the body. PMR improved anxiety and depression ( *P*<0.05), and health-related QoL ( *P*<0.05) for patients with endometriosis in an RCT of 100 women receiving Gonadotrophin-releasing hormone (GnRH) agonist treatment, randomly assigned to 12 weeks of PMR training or a control group. ^47^

Psychological and physical interventions positively impact on QoL in patients with ERPP. However, there remains a lack of high-powered trials in mind and body therapies. Consideration must be taken for the barriers to accessing psychological interventions. Particularly, patients should not feel their pain is less validated if a physiological approach is offered. Nowadays smart-phone applications are often suggested to simplify access to Mindfulness. However, those approaches require co-development with stakeholders to be acceptable and used regularly. ^48^

### Physiotherapy

***Pelvic floor muscle physiotherapy***

Pelvic floor muscle dysfunction (specifically levator ani hypertonia and incomplete relaxation) contributes to ERPP with deep infiltrative endometriosis (DIE). ^49^^–^^51^

Pelvic floor physiotherapy (PFP), assessed by 3D/4D trans-perineal ultrasound, increased levator hiatus area (LHA) which in turn improved dyspareunia and pelvic floor muscle relaxation (PFMR) reduced ERPP. Following a successful pilot study, ^52^ Forno et al used trans-perineal ultrasound to assess LHA before and after PFP in an RCT of 34 women with ERPP. ^53^ Participants were assigned to treatment with five PFP sessions (n=17) or no intervention (n=17). Physiotherapy sessions involved the Thiele massage, using digital pressure to elongate and relax muscles, restoring normal tone. PFMR improved on maximum Valsalva manoeuvre in the intervention group compared to the control (20.0 ± 24.8% *vs* –0.5 ± 3.3%, respectively; *P*=0.02), and superficial dyspareunia pain scores reduced ( *P*<0.01)

### Botulinum toxin

Injection of botulinum toxin may have a role in easing CPP, however existing research lacks standardisation of the toxin brand, site of injection, dose and outcome measures. ^54^

A cohort study of 13 women with endometriosis and pelvic floor spasm reported that 4-8 weeks following injection of 100 units of onabotulinumtoxinA into the pelvic floor muscles reduced pain in all women (median VAS=2, range 0–5/10, *P*<0.0001). ^55^

### Acupuncture

Previous studies have shown acupuncture to be a suitable tool in reducing ERPP, and is considered a safe therapy with minimal side effects. ^56^^,^^57^ Several recent case studies have shown symptomatic improvement with acupuncture. ^58^^,^^59^ Yan et al published a protocol for SR and meta-analysis of RCTs on acupuncture benefits for endometriosis symptoms. ESHRE guidelines ^60^ acknowledge that acupuncture may be a beneficial tool, however the studies that were available at that time were limited and not free from bias.

Wang et al ^61^ recently published a SR of 15 RCTs (sample sizes between 10 and 54), which assessed the effectiveness of acupuncture and/or moxibustion for the treatment of endometriosis. Compared with sham acupuncture, actual acupuncture was more effective at reducing dysmenorrhea VAS pain score (mean difference [MD] − 2.40, 95% CI [− 2.80, − 2.00]; moderate certainty evidence), pelvic pain VAS score (MD − 2.65, 95% CI [− 3.40, − 1.90]; high certainty evidence) and dyspareunia VAS scores (MD − 2.88, [− 3.83, − 1.93]), lessened the size of ovarian cysts (MD − 3.88, 95% CI [− 7.06, − 0.70]), and improved quality of life. These promising results suggest that acupuncture may be an effective adjunct to treating ERPP.

In a multicentre, randomised, single-blind, placebo-controlled trial ^62^ assessing the effects of acupuncture on endometriosis related symptoms (n=106), acupuncture was delivered to the intervention group (n=51) as 30-minute sessions once daily, three times a week, starting one week before expected onset of menstruation, for a total duration of 12-weeks. The control group (n=53) received sham acupuncture. Lower VAS scores were seen in the intervention group at 12 weeks for dysmenorrhoea (-2.82 (-3.47, -2.18) and QoL, (EHP score) -18.88 (-31.88, -5.87)), but not for pelvic pain and dyspareunia. At 24 weeks no statistical benefits were seen, suggesting acupuncture is a suitable immediate therapy for endometriosis related dysmenorrhoea, however the effects of acupuncture may not be sustainable over a long period of time and repeated therapy would be necessary. Notable limitations of this study include the lack of blinding and the inability to assess non-menstrual CPP.

### Adjunct devices

***Phallus length reducing devices***

The Ohnut© device is a phallus length reducer worn over the penis or penetrating object with the intention to reduce endometriosis-associated deep dyspareunia, sparing the cervix and retro-cervical area from direct pressure. The effectiveness of this device is currently being assessed in a pilot RCT of 40 participants with ERPP by Zhang ^63^ who will be randomised into an intervention group or a waitlist control group.

***Transcutaneous electrical nerve stimulation (TENS)***

A TENS unit passes an electrical current through skin electrodes for targeted pain relief. The spinothalamic nerve tract transmits both pain and touch, but not at the same time (gate control theory). ^64^ Its use has been shown to reduce pain in primary dysmenorrhoea ^65^ and CPP. ^66^^,^^67^

Mira et al ^68^ conducted a multicentre RCT of 101 participants with deeply infiltrating endometriosis. The study aimed to identify whether the addition of a TENS unit to hormonal therapy (n=53) would provide a greater therapeutic benefit than hormonal treatment alone (n=48). TENS was used twice a day, 20 minutes per day, for 8 weeks. CPP improved in the intervention group (VAS decreased from 7.11 ± 2.40–4.55 ± 3.08, *P*<0.001, 36% decrease), but not in controls (VAS from 7.33 ± 2.09–7.06 ± 2.33, *P*=0.554, 3.68% decrease). A greater improvement in deep dyspareunia was found in the intervention group, 32.67% reduction vs. 13.84% reduction in the controls. There was a decrease in the number of days participants experienced pain from the first week to the eighth week (from 3.27 to 2.22, *P*=0.028, 32.11% decrease), which was not identified in the control group (from 4.55 to 4.07, *P*=0.203, 10.54% decrease). This study was conducted over a relatively short time interval, therefore due to the chronic nature of endometriosis, further research is needed to assess whether benefits from TENs units are sustained longer-term.

### Cannabinoid (CBT)

CBD has antioxidant, antifibrotic and anti-inflammatory effects, and has been shown to reduce th diameter, volume and area of endometrioma as well as lesion morphology in endometriosis. ^69^ A SR suggests that CBT use can relieve CPP in up to 95.5% of its users. ^70^

A cross sectional survey ^71^ of 113 women with pelvic, perineal pain, dyspareunia or endometriosis was conducted to gather information regarding patient cannabis use. 26/113 (23%) participants reported cannabis use, of which only 5/26 obtained cannabis through a medical programme, 25 had complete data and were analysed. 15/25 (60%) used a combination of CBD and tetrahydrocannabinol (THC). There was no significant difference between the demographics of cannabis users and nonusers. Overall, 24/25 (96%) of participants reported improvement in symptoms such as pain, depression and sleep disturbance with the use of cannabis. It is important to note that participants from both groups also utilised alternative medications and therapies, and therefore reported symptom improvement cannot be confidently solely attributed to cannabis use.

Cannabis use was found to be the most effective form of self-management in an Australian online survey completed by 484 women with endometriosis. Women reported pain relief of 7.6 on a scale of 0-10 with cannabis use (SD 2.0), and 6.3 with hemp oil/CBD oil use (SD 3.0). ^72^

### Traditional Chinese medicine (TCM)

Zhao et al ^73^ performed a non-blinded RCT of 320 patients undergoing endometriosis surgery to investigate the effects of TCM (activating blood circulation and removing blood stasis treatment based on syndrome differentiation; n=131) and Western medicine (GnRH agonist or progesterone’s; n=141) on QOL postoperatively.

Pre-treatment WHOQOL-BREF scores, a QOL assessment tool with four domains including physical health, psychological, social relationships and environment, showed no significant difference between the two groups ( *P*>0.05), however post-treatment scores in the TCM group were significantly improved ( *P*<0.05) and the scores of 4 items (mobility, activities of daily living, sexual activity, QOL score) were also statistically significantly better ( *P*<0.05).

A Cochrane review by Flower et al ^74^ assessed the effects of Chinese herbal medicine (CHM) for endometriosis. Only two RCTs were included (n=158), neither of which compared CHM with placebo. The first showed no significant difference in ERPP between CHM and gestrinone administration following laparoscopic treatment (95.65% vs. 93.87%; risk ratio (RR) 1.02, 95% confidence interval (CI) 0.93 to 1.12, one RCT). Combined oral CHM and herbal enemas provided better improvement in dysmenorrhoea than with danazol (RR 5.06, 95% CI 1.28 to 20.05; RR 5.63, 95% CI 1.47 to 21.54, respectively). There was no significant difference in lumbosacral pain, rectal discomfort, or vaginal nodule tenderness between CHM and danazol. Flower raises concern about the paucity of robust studies assessing on CHM in endometriosis and that the small size of the current studies.

## Discussion

The previous cornerstones of endometriosis care have been shaken. Neuromodulators are less effective than assumed, ^9^ a meaningful proportion do not get pain relief from surgery ^3^ and ⅓ do not respond to progesterone. Complementary, self-management and lifestyle approaches are moving from fringe interest into mainstream endometriosis care. Our review highlights the importance and benefits of integrating these techniques into clinical practice.

A historic RCT ^75^ has shown multimodal holistic approaches yield superior outcomes to early laparoscopy in CPP, but authors of recent SR of five studies (n=186 tertiary centre patients) on interprofessional treatment approaches in CPP criticise the paucity of evidence which does not allow for identification of the best interprofessional treatment approach and call for more research. ^76^

Current UK endometriosis centre accreditation weights bowel surgery heavily but patient education and signposting to holistic evidence-based care is left to enthusiastic HCPs, specialist nurses and patient charities, resulting in care inequities. Accreditation hinges on an MDT of surgeons/urologists, but not with pelvic pain physiotherapists, nutritionists and psychologists.

Numerous calls for more research into complementary approaches need to be answered by appropriate funding.

Within a patient journey, complementary approaches could be used in the following models as a primary approach or in conjunction with routine treatment. ^4^

1. Future women’s health hubs can identify DIE, likely to respond to surgery with specialised scanning (requiring an appropriately trained workforce) even before referral to secondary and tertiary care. Models initiating this is the community would improve patient journeys and shorten the delay in endometriosis patients accessing care.
2. Peri-operatively in the context of pre- and rehabilitation: surgery should no longer be seen in isolation but embedded in education and self-care. It is common knowledge among clinicians that patients recover faster and better from endometriosis surgery if they enter into surgery having practised pre-habilitation.
3. An adjunct to hormonal, surgical and pain-relieving western approaches.
4. In the future, complementary and self-care techniques may be used in prevention of disease recurrence, whereas today the only evidence base is in hormonal manipulation ^77^ but future evidence may enable clinicians to recommend preventive approaches.

## Data Availability

No data are associated with this article.

## References

[1] Smolarz B Szyłło K Romanowicz H : Endometriosis: Epidemiology, Classification, Pathogenesis, Treatment and Genetics (Review of Literature). *Int J Mol Sci.* 2021;22(19). 10.3390/ijms221910554 34638893 PMC8508982

[2] Zheng P Zhang W Leng J : Research on central sensitization of endometriosis-associated pain: a systematic review of the literature. *J Pain Res.* 2019;12:1447–1456. 10.2147/JPR.S197667 31190954 PMC6514255

[3] Singh SS Gude K Perdeaux E : Surgical Outcomes in Patients With Endometriosis: A Systematic Review. *J Obstet Gynaecol Can.* 2020;42(7):881–888.e11. 10.1016/j.jogc.2019.08.004 31718952

[4] Ball E Karavadra B Kremer-Yeatman BJ : Systematic review of patient-specific pre-operative predictors of pain improvement to endometriosis surgery. *Reprod Fertil.* 2021;2(1):69–80. 10.1530/RAF-20-0057 35128434 PMC8812445

[5] Mackenzie SC Stephen J Williams L : Effectiveness of laparoscopic removal of isolated superficial peritoneal endometriosis for the management of chronic pelvic pain in women (ESPriT2): protocol for a multi-centre randomised controlled trial. *Trials.* 2023;24(1):425. 10.1186/s13063-023-07386-x 37349849 PMC10286505

[6] Ianieri MM De Cicco NA Benvenga G : Vascular- and nerve-sparing bowel resection for deep endometriosis: A retrospective single-center study. *Int J Gynaecol Obstet.* 2024;164(1):277–285. 10.1002/ijgo.15019 37555349

[7] Seracchioli R Ferrini G Montanari G : Does laparoscopic shaving for deep infiltrating endometriosis alter intestinal function? A prospective study. *Aust N Z J Obstet Gynaecol.* 2015;55(4):357–362. 10.1111/ajo.12358 26201679

[8] Le Guen M Schantz C Régnier-Loilier A : Reasons for rejecting hormonal contraception in Western countries: A systematic review. *Soc Sci Med.* 2021;284: 114247. 10.1016/j.socscimed.2021.114247 34339927

[9] Vincent K Baranowski A Bhattacharya S : GaPP2, a multicentre randomised controlled trial of the efficacy of gabapentin for the management of chronic pelvic pain in women: study protocol. *BMJ Open.* 2018;8(1): e014924. 10.1136/bmjopen-2016-014924 29391360 PMC5879736

[10] Birkinshaw H Friedrich CM Cole P : Antidepressants for pain management in adults with chronic pain: a network meta-analysis. *Cochrane Database Syst Rev.* 2023;5(5):CD014682. 10.1002/14651858.CD014682.pub2 37160297 PMC10169288

[11] Koirala B Peeler A Dennison Himmelfarb C : Living with multiple chronic conditions: How we achieve holistic care and optimize health outcomes. *J Adv Nurs.* 2023;79(2):e7–e9. 10.1111/jan.15433 36062872 PMC9877113

[12] Field R Pourkazemi F Turton J : Dietary Interventions Are Beneficial for Patients with Chronic Pain: A Systematic Review with Meta-Analysis. *Pain Med.* 2021;22(3):694–714. 10.1093/pm/pnaa378 33202007

[13] Genel F Kale M Pavlovic N : Health effects of a low-inflammatory diet in adults with arthritis: a systematic review and meta-analysis. *J Nutr Sci.* 2020;9: e37. 10.1017/jns.2020.31 32983422 PMC7503186

[14] Brain K Burrows TL Bruggink L : Diet and Chronic Non-Cancer Pain: The State of the Art and Future Directions. *J Clin Med.* 2021;10(21). 10.3390/jcm10215203 34768723 PMC8584994

[15] Ustianowska K Ustianowski Ł Machaj F : The Role of the Human Microbiome in the Pathogenesis of Pain. *Int J Mol Sci.* 2022;23(21). 10.3390/ijms232113267 36362056 PMC9659276

[16] Nirgianakis K Egger K Kalaitzopoulos DR : Effectiveness of Dietary Interventions in the Treatment of Endometriosis: a Systematic Review. *Reprod Sci.* 2022;29(1):26–42. 10.1007/s43032-020-00418-w 33761124 PMC8677647

[17] Cobellis L Castaldi MA Giordano V : Effectiveness of the association micronized N-Palmitoylethanolamine (PEA)-transpolydatin in the treatment of chronic pelvic pain related to endometriosis after laparoscopic assessment: a pilot study. *Eur J Obstet Gynecol Reprod Biol.* 2011;158(1):82–86. 10.1016/j.ejogrb.2011.04.011 21601979

[18] Giugliano E Cagnazzo E Soave I : The adjuvant use of N-palmitoylethanolamine and transpolydatin in the treatment of endometriotic pain. *Eur J Obstet Gynecol Reprod Biol.* 2013;168(2):209–213. 10.1016/j.ejogrb.2013.01.009 23415738

[19] Indraccolo U Barbieri F : Effect of palmitoylethanolamide-polydatin combination on chronic pelvic pain associated with endometriosis: preliminary observations. *Eur J Obstet Gynecol Reprod Biol.* 2010;150(1):76–79. 10.1016/j.ejogrb.2010.01.008 20176435

[20] Sverrisdóttir U Hansen S Rudnicki M : Impact of diet on pain perception in women with endometriosis: A systematic review. *Eur J Obstet Gynecol Reprod Biol.* 2022;271:245–249. 10.1016/j.ejogrb.2022.02.028 35245715

[21] Moore JS Gibson PR Perry RE : Endometriosis in patients with irritable bowel syndrome: Specific symptomatic and demographic profile, and response to the low FODMAP diet. *Aust N Z J Obstet Gynaecol.* 2017;57(2):201–205. 10.1111/ajo.12594 28303579

[22] Nabi MY Nauhria S Reel M : Endometriosis and irritable bowel syndrome: A systematic review and meta-analyses. *Front Med (Lausanne).* 2022;9: 914356. 10.3389/fmed.2022.914356 35957857 PMC9357916

[23] Velho RV Werner F Mechsner S : Endo Belly: What Is It and Why Does It Happen?-A Narrative Review. *J Clin Med.* 2023;12(22):7176. 10.3390/jcm12227176 38002788 PMC10671958

[24] Yuk JS Shin JS Shin JY : Nickel Allergy Is a Risk Factor for Endometriosis: An 11-Year Population-Based Nested Case-Control Study. *PLoS One.* 2015;10(10): e0139388. 10.1371/journal.pone.0139388 26439741 PMC4594920

[25] Borghini R Porpora MG Casale R : Irritable Bowel Syndrome-Like Disorders in Endometriosis: Prevalence of Nickel Sensitivity and Effects of a Low-Nickel Diet. An Open-Label Pilot Study. *Nutrients.* 2020;12(2). 10.3390/nu12020341 32012984 PMC7071203

[26] Krabbenborg I Roos N de Grinten P van der : Diet quality and perceived effects of dietary changes in Dutch endometriosis patients: an observational study. *Reprod Biomed Online.* 2021;43(5):952–961. 10.1016/j.rbmo.2021.07.011 34493462

[27] Amirsalari S Behboodi Moghadam Z Taghizadeh Z : The Effect of Garlic Tablets on the Endometriosis-Related Pains: A Randomized Placebo-Controlled Clinical Trial. *Evid Based Complement Alternat Med.* 2021;2021:1–8. 10.1155/2021/5547058 PMC8315864 34335819

[28] Liu Y Zhang W : Association between body mass index and endometriosis risk: a meta-analysis. *Oncotarget.* 2017;8(29):46928–46936. 10.18632/oncotarget.14916 28159926 PMC5564533

[29] Hong J Yi KW : What is the link between endometriosis and adiposity? *Obstet Gynecol Sci.* 2022;65(3):227–233. 10.5468/ogs.21343 35081675 PMC9119730

[30] Netzl J Gusy B Voigt B : Chronic Pelvic Pain in Endometriosis: Cross-Sectional Associations with Mental Disorders, Sexual Dysfunctions and Childhood Maltreatment. *J Clin Med.* 2022;11(13). 10.3390/jcm11133714 35807000 PMC9267229

[31] McPeak AE Allaire C Williams C : Pain Catastrophizing and Pain Health-Related Quality-of-Life in Endometriosis. *Clin J Pain.* 2018;34(4):349–356. 10.1097/AJP.0000000000000539 28731958

[32] Carey ET Martin CE Siedhoff MT : Biopsychosocial correlates of persistent postsurgical pain in women with endometriosis. *Int J Gynaecol Obstet.* 2014;124(2):169–173. 10.1016/j.ijgo.2013.07.033 24290537

[33] Gholiof M Adamson-De Luca E Foster WG : Prevalence of Use and Perceived Effectiveness of Medical, Surgical, and Alternative Therapies for Endometriosis Pain in Canadians. *J Obstet Gynaecol Can.* 2023;45(1):11–20. 10.1016/j.jogc.2022.11.003 36455861

[34] Brooks T Sharp R Evans S : Psychological Interventions for Women with Persistent Pelvic Pain: A Survey of Mental Health Clinicians. *J Multidiscip Healthc.* 2021;14:1725–1740. 10.2147/JMDH.S313109 34262286 PMC8275108

[35] Schubert K Lohse J Kalder M : Internet-based cognitive behavioral therapy for improving health-related quality of life in patients with endometriosis: study protocol for a randomized controlled trial. *Trials.* 2022;23(1):300. 10.1186/s13063-022-06204-0 35414092 PMC9006397

[36] Boersen Z Oosterman J Hameleers EG : Determining the effectiveness of cognitive behavioural therapy in improving quality of life in patients undergoing endometriosis surgery: a study protocol for a randomised controlled trial. *BMJ Open.* 2021;11(12): e054896. 10.1136/bmjopen-2021-054896 34880026 PMC8655560

[37] Mikocka-Walus A Druitt M O’Shea M : Yoga, cognitive-behavioural therapy versus education to improve quality of life and reduce healthcare costs in people with endometriosis: a randomised controlled trial. *BMJ Open.* 2021;11(8): e046603. 10.1136/bmjopen-2020-046603 34373298 PMC8354255

[38] Wu S Wang X Liu H : Efficacy of cognitive behavioral therapy after the surgical treatment of women with endometriosis: A preliminary case-control study. *Medicine (Baltimore).* 2022;101(51): e32433. 10.1097/MD.0000000000032433 36595829 PMC9794269

[39] Noonan S : Mindfulness-based stress reduction. *Can Vet J.* 2014;55(2):134–135. 24489390 PMC3894868

[40] Moreira MF Gamboa OL Pinho Oliveira MA : A single-blind, randomized, pilot study of a brief mindfulness-based intervention for the endometriosis-related pain management. *Eur J Pain.* 2022;26(5):1147–1162. 10.1002/ejp.1939 35276031

[41] Hansen KE Brandsborg B Kesmodel US : Psychological interventions improve quality of life despite persistent pain in endometriosis: results of a 3-armed randomized controlled trial. *Qual Life Res.* 2023;32(6):1727–1744. 10.1007/s11136-023-03346-9 36797461 PMC10172241

[42] Ravins I Joseph G Tene L : The Effect of Practicing “Endometriosis Yoga” on Stress and Quality of Life for Women with Endometriosis: AB Design Pilot Study. *Altern Ther Health Med.* 2023;29(3):8–14. 35839113

[43] Gonçalves AV Barros NF Bahamondes L : The Practice of Hatha Yoga for the Treatment of Pain Associated with Endometriosis. *J Altern Complement Med.* 2017;23(1):45–52. 10.1089/acm.2015.0343 27869485

[44] Saxena R Gupta M Shankar N : Effects of yogic intervention on pain scores and quality of life in females with chronic pelvic pain. *Int J Yoga.* 2017;10(1):9–15. 10.4103/0973-6131.186155 28149062 PMC5225749

[45] Yin B Jiang H Liu X : Enriched Environment Decelerates the Development of Endometriosis in Mouse. *Reprod Sci.* 2020;27(7):1423–1435. 10.1007/s43032-019-00117-1 32318984

[46] F I B-C B DH G : *Impact of Environmental Enrichment on Pain, Perceived Stress, Mental Health and Quality of Life of Women with Endometriosis The World Congress on Endometriosis.* Edinburgh, UK;2023.

[47] Zhao L Wu H Zhou X : Effects of progressive muscular relaxation training on anxiety, depression and quality of life of endometriosis patients under gonadotrophin-releasing hormone agonist therapy. *Eur J Obstet Gynecol Reprod Biol.* 2012;162(2):211–215. 10.1016/j.ejogrb.2012.02.029 22455972

[48] Ball E Rivas C : Health Apps Require Co-development to Be Acceptable and Effective. *Front Psychol.* 2021;12: 714453. 10.3389/fpsyg.2021.714453 34335428 PMC8322940

[49] Fraga MV Oliveira Brito LG Yela DA : Pelvic floor muscle dysfunctions in women with deep infiltrative endometriosis: An underestimated association. *Int J Clin Pract.* 2021;75(8): e14350. 10.1111/ijcp.14350 33973308

[50] Silva JP da Almeida BM de Ferreira RS : Sensory and muscular functions of the pelvic floor in women with endometriosis - cross-sectional study. *Arch Gynecol Obstet.* 2023;308(1):163–170. 10.1007/s00404-023-07037-1 37042996

[51] Arena A Degli Esposti E Cocchi L : Three-Dimensional Ultrasound Evaluation of Pelvic Floor Muscle Contraction in Women Affected by Deep Infiltrating Endometriosis: Application of a Quick Contraction Scale. *J Ultrasound Med.* 2022;41(12):2973–2979. 10.1002/jum.15996 35532292

[52] Del Forno S Arena A Alessandrini M : Transperineal Ultrasound Visual Feedback Assisted Pelvic Floor Muscle Physiotherapy in Women With Deep Infiltrating Endometriosis and Dyspareunia: A Pilot Study. *J Sex Marital Ther.* 2020;46(7):603–611. 10.1080/0092623X.2020.1765057 32579077

[53] Del Forno S Arena A Pellizzone V : Assessment of levator hiatal area using 3D/4D transperineal ultrasound in women with deep infiltrating endometriosis and superficial dyspareunia treated with pelvic floor muscle physiotherapy: randomized controlled trial. *Ultrasound Obstet Gynecol.* 2021;57(5):726–732. 10.1002/uog.23590 33428320

[54] Karp BI Stratton P : Applications of botulinum toxin to the female pelvic floor: Botulinum toxin for genito-pelvic pain penetration disorder and chronic pelvic pain in women. *Toxicon.* 2023;230: 107162. 10.1016/j.toxicon.2023.107162 37201800 PMC10330736

[55] Tandon HK Stratton P Sinaii N : Botulinum toxin for chronic pelvic pain in women with endometriosis: a cohort study of a pain-focused treatment. *Reg Anesth Pain Med.* 10.1136/rapm-2019-100529 PMC6946887 31289238

[56] Mira TAA Buen MM Borges MG : Systematic review and meta-analysis of complementary treatments for women with symptomatic endometriosis. *Int J Gynaecol Obstet.* 2018;143(1):2–9. 10.1002/ijgo.12576 29944729

[57] Payne JA : Acupuncture for Endometriosis: A Case Study. *Med Acupunct.* 2019;31:392–394. United States: Copyright 2019, Mary Ann Liebert, Inc., publishers. 10.1089/acu.2019.1379 31871528 PMC6918512

[58] Martin BR : Multimodal Care for Headaches, Lumbopelvic Pain, and Dysmenorrhea in a Woman With Endometriosis: A Case Report. *J Chiropr Med.* 2021;20:148–157. United States: © 2022 by National University of Health Sciences. 10.1016/j.jcm.2021.10.002 35463839 PMC9023129

[59] Yan Q Li J Zeng J : The role of acupuncture in the treatment of women with pain in endometriosis: A protocol for systematic review and meta-analysis. *Medicine (Baltimore).* 2021;100(49): e27582. 10.1097/MD.0000000000027582 34889220 PMC8663820

[60] Becker CM Bokor A Heikinheimo O : ESHRE guideline: endometriosis. *Hum Reprod Open.* 2022;2022(2):hoac009. 10.1093/hropen/hoac009 35350465 PMC8951218

[61] Wang Y Coyle ME Hong M : Acupuncture and moxibustion for endometriosis: A systematic review and analysis. *Complement Ther Med.* 2023;76: 102963. 10.1016/j.ctim.2023.102963 37453585

[62] Li PS Peng XM Niu XX : Efficacy of acupuncture for endometriosis-associated pain: a multicenter randomized single-blind placebo-controlled trial. *Fertil Steril.* 2023;119(5):815–823. 10.1016/j.fertnstert.2023.01.034 36716811

[63] Zhang SXJ MacLeod RGK Parmar G : Ohnut Versus a Waitlist Control for the Self-management of Endometriosis-Associated Deep Dyspareunia: Protocol for a Pilot Randomized Controlled Trial. *JMIR Res Protoc.* 2023;12: e39834. 10.2196/39834 36972117 PMC10131731

[64] Teoli D An J : Transcutaneous Electrical Nerve Stimulation. StatPearls.Treasure Island (FL): StatPearls Publishing Copyright © 2023, StatPearls Publishing LLC;2023. 30725873

[65] Arik MI Kiloatar H Aslan B : The effect of TENS for pain relief in women with primary dysmenorrhea: A systematic review and meta-analysis. *Explore (NY).* 2022;18(1):108–113. 10.1016/j.explore.2020.08.005 32917532

[66] Bridger C Prabhala T Dawson R : Neuromodulation for Chronic Pelvic Pain: A Single-Institution Experience With a Collaborative Team. *Neurosurgery.* 2021;88(4):819–827. 10.1093/neuros/nyaa537 33372201 PMC7956019

[67] Guy M Foucher C Juhel C : Transcutaneous electrical neurostimulation relieves primary dysmenorrhea: A randomized, double-blind clinical study versus placebo. *Prog Urol.* 2022;32(7):487–497. 10.1016/j.purol.2022.01.005 35249825

[68] Mira TAA Yela DA Podgaec S : Hormonal treatment isolated versus hormonal treatment associated with electrotherapy for pelvic pain control in deep endometriosis: Randomized clinical trial. *Eur J Obstet Gynecol Reprod Biol.* 2020;255:134–141. 10.1016/j.ejogrb.2020.10.018 33129015

[69] Genovese T Cordaro M Siracusa R : Molecular and Biochemical Mechanism of Cannabidiol in the Management of the Inflammatory and Oxidative Processes Associated with Endometriosis. *Int J Mol Sci.* 2022;23(10):5427. 10.3390/ijms23105427 35628240 PMC9141153

[70] Liang AL Gingher EL Coleman JS : Medical Cannabis for Gynecologic Pain Conditions: A Systematic Review. *Obstet Gynecol.* 2022;139(2):287–296. 10.1097/AOG.0000000000004656 35104069

[71] Carrubba AR Ebbert JO Spaulding AC : Use of Cannabis for Self-Management of Chronic Pelvic Pain. *J Womens Health (Larchmt).* 2021;30(9):1344–1351. 10.1089/jwh.2020.8737 33252316

[72] Armour M Sinclair J Chalmers KJ : Self-management strategies amongst Australian women with endometriosis: a national online survey. *BMC Complement Altern Med.* 2019;19(1):17. 10.1186/s12906-019-2431-x 30646891 PMC6332532

[73] Zhao RH Liu Y Tan Y : Chinese medicine improves postoperative quality of life in endometriosis patients: a randomized controlled trial. *Chin J Integr Med.* 2013;19(1):15–21. 10.1007/s11655-012-1196-6 23275012

[74] Flower A Liu JP Lewith G : Chinese herbal medicine for endometriosis. *Cochrane Database Syst Rev.* 2012;2012(5):CD006568. 10.1002/14651858.CD006568.pub3 PMC12817023 22592712

[75] Peters AA Dorst E van Jellis B : A randomized clinical trial to compare two different approaches in women with chronic pelvic pain. *Obstet Gynecol.* 1991;77(5):740–744. 1826544

[76] Klotz SGR Kolbe C Rueß M : The role of psychosocial factors in the interprofessional management of women with chronic pelvic pain: A systematic review. *Acta Obstet Gynecol Scand.* 2024;103(2):199–209. 10.1111/aogs.14708 37961843 PMC10823391

[77] Zheng Q Mao H Xu Y : Can postoperative GnRH agonist treatment prevent endometriosis recurrence? *A meta-analysis. Arch Gynecol Obstet.* 2016;294(1):201–207. 10.1007/s00404-016-4085-y 27052442

