# Supplementary material for: Holistic approaches to living well with endometriosis
Source: F1000Res. 2024 Nov 8;13:359. Originally published 2024 Apr 23. [Version 2] doi: 10.12688/f1000research.142586.2 (PMC11621616; doi:10.12688/f1000research.142586.2)
Supplement: Peer review history

# Reviewer report: Reviewer response for version 2

**DOI:** 10.5256/f1000research.174168.r338887

**Published:** 2024-12-05

**Authors:** D Raimondo

**Affiliations:** 1 Division of Gynaecology and Human Reproduction Physiopathology, IRCCS AOUBO, Bologna, Italy

auhtors replied to all queries

Is the review written in accessible language?

Yes

Are all factual statements correct and adequately supported by citations?

Yes

Are the conclusions drawn appropriate in the context of the current research literature?

Yes

Is the topic of the review discussed comprehensively in the context of the current literature?

Yes

Reviewer Expertise:

endometriosis

I confirm that I have read this submission and believe that I have an appropriate level of expertise to confirm that it is of an acceptable scientific standard.

# Reviewer report: Reviewer response for version 2

**DOI:** 10.5256/f1000research.174168.r338886

**Published:** 2024-11-18

**Authors:** Philippa Saunders

**Affiliations:** 1 MRC Centre for Reproductive Health, University of Edinburgh, Edinburgh, UK

The authors have improved the balance so it can be moved into the approved category.

Is the review written in accessible language?

Yes

Are all factual statements correct and adequately supported by citations?

No

Are the conclusions drawn appropriate in the context of the current research literature?

No

Is the topic of the review discussed comprehensively in the context of the current literature?

No

Reviewer Expertise:

Reproductive biologist, specialising in women's health conditions including endometriosis. Translational research focused on improving range of treatments for chronic pelvic pain including self management strategies.

I confirm that I have read this submission and believe that I have an appropriate level of expertise to confirm that it is of an acceptable scientific standard.

# Reviewer report: Reviewer response for version 1

**DOI:** 10.5256/f1000research.156153.r292395

**Published:** 2024-07-22

**Authors:** Philippa Saunders

**Affiliations:** 1 MRC Centre for Reproductive Health, University of Edinburgh, Edinburgh, UK

Endometriosis is an incurable disorder and as the authors correctly state patients are often keen to try non-medical approaches to manage symptoms including chronic pain, bloating and fatigue. Some approaches including physiotherapy are often included in multidisciplinary care and information on patient websites includes a wide range of complementary therapies some of which are discussed in this review [ https://www.endometriosis-uk.org/].

 The authors are to be commended on bringing together information about a very wide range of potential interventions and their assessment that many individuals with symptomatic endometriosis are keen to explore self management and alternative therapies as part of their care plan is something increasingly reported by health care professionals.

 The section entitled ‘Recent findings’ makes some dogmatic statements and it was not clear  they were backed up by the latest data – this was particularly true of nutrition which is a fast moving field.

 Given the importance of the topic it is of concern that there appears to be an overall lack of ‘balance’ in the  paper with a lot of emphasis in the first section placed on a systematic reviews – REFs 1-5, 8,9,12.

 In some parts of the text it is hard to know if the results discussed relate to individuals with CPP or those with CPP/Endo so careful re-reading and clarification of is advised (examples include yoga, TENS, mindfulness). [table of mind and body therapies?]

 It is recommended that the authors assemble information from relevant high quality original information from trials in tables – this would be more useful to the reader than simple text. Authors are referred to a number of recent reviews related to the impact of diet, inflammation and the gut brain axis as sources of information.

 Additional References

 Esprit2 trial has a published protocol [Ref -1].

 Reference 6 is to a protocol – the trial has been completed and the correct reference is

 [Ref -2]

 In section on nutrition it would be useful to mention GWAS and other data that support patient experience of IBS-like symptoms in association with endometriosis (includes abdominal bloating [Ref-3], diarrhoea etc).

 Recent paper [Ref-3]

 No mention of botox for treatment of pelvic floor pain although there are reports it is effective · [Ref -4]

 The section on cannabinoids is very brief which seems at odds with the widespread use of CBD in management of chronic pain, in some countries they are prescribed and there are several trials for endometriosis CPP underway – add more references. [Ref 5-7]

 Add more details from Ref 64 – this paper is from 1991 – any follow up papers?

Is the review written in accessible language?

Yes

Are all factual statements correct and adequately supported by citations?

No

Are the conclusions drawn appropriate in the context of the current research literature?

No

Is the topic of the review discussed comprehensively in the context of the current literature?

No

Reviewer Expertise:

Reproductive biologist, specialising in women's health conditions including endometriosis. Translational research focused on improving range of treatments for chronic pelvic pain including self management strategies.

I confirm that I have read this submission and believe that I have an appropriate level of expertise to state that I do not consider it to be of an acceptable scientific standard, for reasons outlined above.

## References

[1] : Effectiveness of laparoscopic removal of isolated superficial peritoneal endometriosis for the management of chronic pelvic pain in women (ESPriT2): protocol for a multi-centre randomised controlled trial. *Trials* .2023;24(1) : 10.1186/s13063-023-07386-x 425 10.1186/s13063-023-07386-x 37349849 PMC10286505

[2] : Gabapentin for chronic pelvic pain in women (GaPP2): a multicentre, randomised, double-blind, placebo-controlled trial. *Lancet* .2020;396(10255) : 10.1016/S0140-6736(20)31693-7 909-917 10.1016/S0140-6736(20)31693-7 32979978 PMC7527829

[3] : Endo Belly: What Is It and Why Does It Happen?-A Narrative Review. *J Clin Med* .2023;12(22) : 10.3390/jcm12227176 10.3390/jcm12227176 38002788 PMC10671958

[4] : Applications of botulinum toxin to the female pelvic floor: Botulinum toxin for genito-pelvic pain penetration disorder and chronic pelvic pain in women. *Toxicon* .2023;230: 10.1016/j.toxicon.2023.107162 107162 10.1016/j.toxicon.2023.107162 37201800 PMC10330736

[5] : Molecular and Biochemical Mechanism of Cannabidiol in the Management of the Inflammatory and Oxidative Processes Associated with Endometriosis. *Int J Mol Sci* .2022;23(10) : 10.3390/ijms23105427 10.3390/ijms23105427 35628240 PMC9141153

[6] : Medical Cannabis for Gynecologic Pain Conditions: A Systematic Review. *Obstet Gynecol* .2022;139(2) : 10.1097/AOG.0000000000004656 287-296 10.1097/AOG.0000000000004656 35104069

[7] : Self-management strategies amongst Australian women with endometriosis: a national online survey. *BMC Complement Altern Med* .2019;19(1) : 10.1186/s12906-019-2431-x 17 10.1186/s12906-019-2431-x 30646891 PMC6332532

# Response

**Published:** 2024-10-28

**Authors:** Jessica Desai

**Affiliations:** Central and North West London NHS Foundation Trust, London, England, UK

We thank you for the suggestions and have made the following adjustments:

 In some parts of the text it is hard to know if the results discussed relate to individuals with CPP or those with CPP/Endo so careful re-reading and clarification of is advised (examples include yoga, TENS, mindfulness). [table of mind and body therapies?]

 - **Clarification has been made on relation to CPP/endometriosis throughout the text**

 It is recommended that the authors assemble information from relevant high quality original information from trials in tables – this would be more useful to the reader than simple text.

 - **Please see Table 1**

 Esprit2 trial has a published protocol [Ref -1].

 - **Espirit2 trial protocol has now been referenced, please see reference 5**

 Reference 6 is to a protocol – the trial has been completed and the correct reference is

 [Ref -2]

 - **Reference amended**

 In section on nutrition it would be useful to mention GWAS and other data that support patient experience of IBS-like symptoms in association with endometriosis (includes abdominal bloating [Ref-3], diarrhoea etc).

 Recent paper [Ref-3]

 - **Please see use of reference 23 in our paper**

 No mention of botox for treatment of pelvic floor pain although there are reports it is effective · [Ref -4]

 - **Section on botulinum toxin added to paper**

 The section on cannabinoids is very brief which seems at odds with the widespread use of CBD in management of chronic pain, in some countries they are prescribed and there are several trials for endometriosis CPP underway – add more references. [Ref 5-7]

 - **CBD section has been expanded, please see references 69, 70**

 Add more details from Ref 64 – this paper is from 1991 – any follow up papers?

 - **Section expanded, please refer to reference 76**

# Reviewer report: Reviewer response for version 1

**DOI:** 10.5256/f1000research.156153.r271069

**Published:** 2024-07-02

**Authors:** D Raimondo

**Affiliations:** 1 Division of Gynaecology and Human Reproduction Physiopathology, IRCCS AOUBO, Bologna, Italy

Abstract:

 The abstract is well-structured and provides a clear overview of the topic. However, it could benefit from a concluding sentence that highlights the clinical significance of the findings to enhance reader understanding.

 Introduction:

 The introduction is comprehensive and provides adequate context on endometriosis and the challenges associated with its treatment. However, it could benefit from emphasizing more recent research, particularly from the last two to three years.

 Please discuss on the iatrogenic risk of endometriosis surgery and not only on the risk of recurrence (i.e. Ref [1,2])

 Methods:

 The methodology section is detailed and rigorous but could use some clarifications.

**Inclusion and Exclusion Criteria:** Provide more details on the criteria used to include or exclude specific studies.

**Search Methodology:** Describe the databases used and the search terms applied more specifically.

**Quality Assessment:** Describe the metrics or tools used to evaluate the quality of the included studies.

 Results:

 The results are well-presented and supported by a solid evidence base. However, some sections could benefit from more synthesis to improve readability.

**Nutrition:** The information is detailed and pertinent, but a summary table could help visualize the effects of different dietary interventions better.

**Acupuncture:** The acupuncture section is thorough but could benefit from a discussion on the limitations of the cited studies.

 Conclusions:

 The conclusions effectively summarize the key points of the article. However, reinforcing the message on the importance of integrating holistic strategies into clinical practice could enhance the impact.

**Graphs and Tables:** Including more graphs and tables to visualize key data could improve the manuscript's comprehensibility and visual appeal..

Is the review written in accessible language?

Yes

Are all factual statements correct and adequately supported by citations?

Yes

Are the conclusions drawn appropriate in the context of the current research literature?

Yes

Is the topic of the review discussed comprehensively in the context of the current literature?

Yes

Reviewer Expertise:

endometriosis

I confirm that I have read this submission and believe that I have an appropriate level of expertise to confirm that it is of an acceptable scientific standard, however I have significant reservations, as outlined above.

## References

[1] : Does laparoscopic shaving for deep infiltrating endometriosis alter intestinal function? A prospective study. *Aust N Z J Obstet Gynaecol* .2015;55(4) : 10.1111/ajo.12358 357-62 10.1111/ajo.12358 26201679

[2] : Vascular- and nerve-sparing bowel resection for deep endometriosis: A retrospective single-center study. *Int J Gynaecol Obstet* .2024;164(1) : 10.1002/ijgo.15019 277-285 10.1002/ijgo.15019 37555349

# Response

**Published:** 2024-10-28

**Authors:** Jessica Desai

**Affiliations:** Central and North West London NHS Foundation Trust, London, England, UK

We thank you for the suggestions and have made the following adjustments to our paper: 

 Abstract:

 The abstract is well-structured and provides a clear overview of the topic. However, it could benefit from a concluding sentence that highlights the clinical significance of the findings to enhance reader understanding.

  - **Abstract concluding sentence adjusted**

 Introduction:

 The introduction is comprehensive and provides adequate context on endometriosis and the challenges associated with its treatment. However, it could benefit from emphasizing more recent research, particularly from the last two to three years.

 Please discuss on the iatrogenic risk of endometriosis surgery and not only on the risk of recurrence (i.e. Ref [1,2])

 - **Please refer to reference 6 and 7 cited in the amended submission**

 Methods:

 The methodology section is detailed and rigorous but could use some clarifications.

 Inclusion and Exclusion Criteria: Provide more details on the criteria used to include or exclude specific studies.

 Search Methodology: Describe the databases used and the search terms applied more specifically.

 Quality Assessment: Describe the metrics or tools used to evaluate the quality of the included studies.

 - **Inclusion and exclusion criteria, search methodology and quality assessment sections added to paper**

 Results:

 The results are well-presented and supported by a solid evidence base. However, some sections could benefit from more synthesis to improve readability.

 Nutrition: The information is detailed and pertinent, but a summary table could help visualize the effects of different dietary interventions better.

 Acupuncture: The acupuncture section is thorough but could benefit from a discussion on the limitations of the cited studies.

 - **Limitations of paper reference 62 has been added**

 Conclusions:

 The conclusions effectively summarize the key points of the article. However, reinforcing the message on the importance of integrating holistic strategies into clinical practice could enhance the impact.

 - **Conclusion has been amended with statement on the importance of integration into clinical practice**

 Graphs and Tables: Including more graphs and tables to visualize key data could improve the manuscript's comprehensibility and visual appeal..

 - **Please refer to Table 1**
